# Insufficient referral practices of sick children in Ethiopia shown in a cross‐sectional survey

**DOI:** 10.1111/apa.15200

**Published:** 2020-02-17

**Authors:** Habtamu Beyene, Dejene Hailu, Henok Tadele, Lars Åke Persson, Della Berhanu

**Affiliations:** ^1^ Southern Nations, Nationalities & Peoples Regional Health Bureau Hawassa Ethiopia; ^2^ College of Medicine and Health Sciences Hawassa University Hawassa Ethiopia; ^3^ Department of Paediatrics and Child Health College of Health Sciences Addis Ababa University Addis Ababa Ethiopia; ^4^ London School of Hygiene & Tropical Medicine London UK

**Keywords:** health extension workers, logistics, primary health care, referral, sick child

## Abstract

**Aim:**

This study aimed at assessing the referral of sick young infants and children from the community, health posts and health centres to higher levels.

**Methods:**

A cross‐sectional survey was conducted in four of the largest Ethiopian regions from December 2016 to February 2017. Referral practices were assessed at each level in 46 districts of these regions. Interviews were supplemented by reviews of registers at health posts and health centres.

**Results:**

The women's development group leaders, who do not provide health services, referred half of the sick children they visited in the community to the health posts. The health extension workers referred 16% of the sick young infants and 6% of older infants and children to higher levels. From health centres, the health workers referred 6% of sick young infants and 1% of older infants and children to hospital. Many cases of possible severe bacterial infection were not referred to higher levels. A functional ambulance was available for a bit more than a third of the health centres.

**Conclusion:**

Referral practices of sick young infants and children at all levels were weak that may threaten the continued reduction of child mortality in Ethiopia. Referral logistics were insufficient, which partly could explain the missing referrals of severely ill infants and children.

**Key notes**

- An effective referral system for severely ill infants and children is needed for a well‐functioning primary care system.
- We found that referral practices in Ethiopia were weak from community to health posts, from health posts to health centres, and from health centres to hospitals including few referrals even for severe bacterial infections.
- The culture and logistics of referral of severely ill infants need improvement for further reduction of child mortality.

## 1 INTRODUCTION

An estimated 5.4 million children below 5 years of age died in 2017. This figure included 2.4 million neonatal deaths. Most of these deaths occurred in sub‐Saharan Africa and Southern Asia.^1^

Ethiopia reached the 4th Millennium Development Goal of reducing under‐five mortality by two‐thirds.^2^ The 2019 Ethiopian Mini Demographic and Health Survey reported an under‐five mortality rate of 55 deaths per 1000 live births, and a neonatal mortality rate of 30 deaths per 1000 live births.^3^

Most under‐five deaths were caused by diseases that are readily preventable or treatable with proven, cost‐effective interventions if delivered with quality. The Integrated Management of Neonatal and Childhood Illnesses aims to reduce under‐five mortality.^4^ In Ethiopia, this programme was introduced in 1997, with services provided by health officers and nurses at the health centre.^5^ Similarly, the integrated Community Case Management of childhood illnesses is a community‐level strategy to increase access to effective case management for young children suffering from malaria, pneumonia and diarrhoea, especially in hard‐to‐reach and vulnerable populations.^6^, ^7^

The integrated Community Case Management programme was initiated in 2010, using the existing Health Extension Programme as a platform. Ethiopia's Health Extension Programme is a community‐based strategy to deliver health promotion, disease prevention and selected curative health services at the community level. It was launched in 2003 and had 16 packages with a specific focus on women and children.^8^ This programme is delivered through health extension workers, who are salaried government employees working at the health posts and in the community. They are trained to assess, classify and treat children with pneumonia, diarrhoea and malnutrition and to refer severe cases to health centres following guidelines.^7^, ^9^ Ethiopia has also implemented the Community‐Based Newborn Care programme. This programme allows health extension workers to treat young infants (0‐59 days) with possible severe bacterial infection with antibiotics in a pre‐referral dose or with a full‐treatment course when the referral is not possible.^10^

The provision of these infant and child health services at health posts and health centres is supported by laywomen volunteers, known as the Women's Development Group leaders, who promote the utilisation of maternal, newborn and child health services. They also refer pregnant women and sick infants and children to health facilities.^11^

An effective referral system is required to ensure adequate and timely treatment, optimise utilisation, and to coordinate available services and resources.^12^ A functional referral system can only be created if the healthcare providers can identify the severely sick child that needs a referral, if caretakers can comply with the referral, and if the higher‐level health facility receives and appropriately treats the referred children.^13^

Despite all the importance of the implemented programmes in saving the lives of under‐five children, the referral systems in low‐income countries implementing these programmes have been criticised for being weak.^14^ Ethiopia also faces the challenges of a weak referral system, which might contribute to the current level of under‐five mortality.^3^

Few previous studies have analysed sick child referrals across the health system levels in Ethiopia. This study aimed at assessing referral of sick infants and children from the community, health posts and health centres to higher levels. We also evaluated the knowledge of referral guidelines among health workers and the available logistics and infrastructure that could support referrals.

## 2 METHODS

### 2.1 Design and study area

A baseline cross‐sectional survey was conducted before the implementation of optimising the health extension programme intervention, which aimed at improving the utilisation of community‐based child health services in Ethiopia.

**Figure 1:**
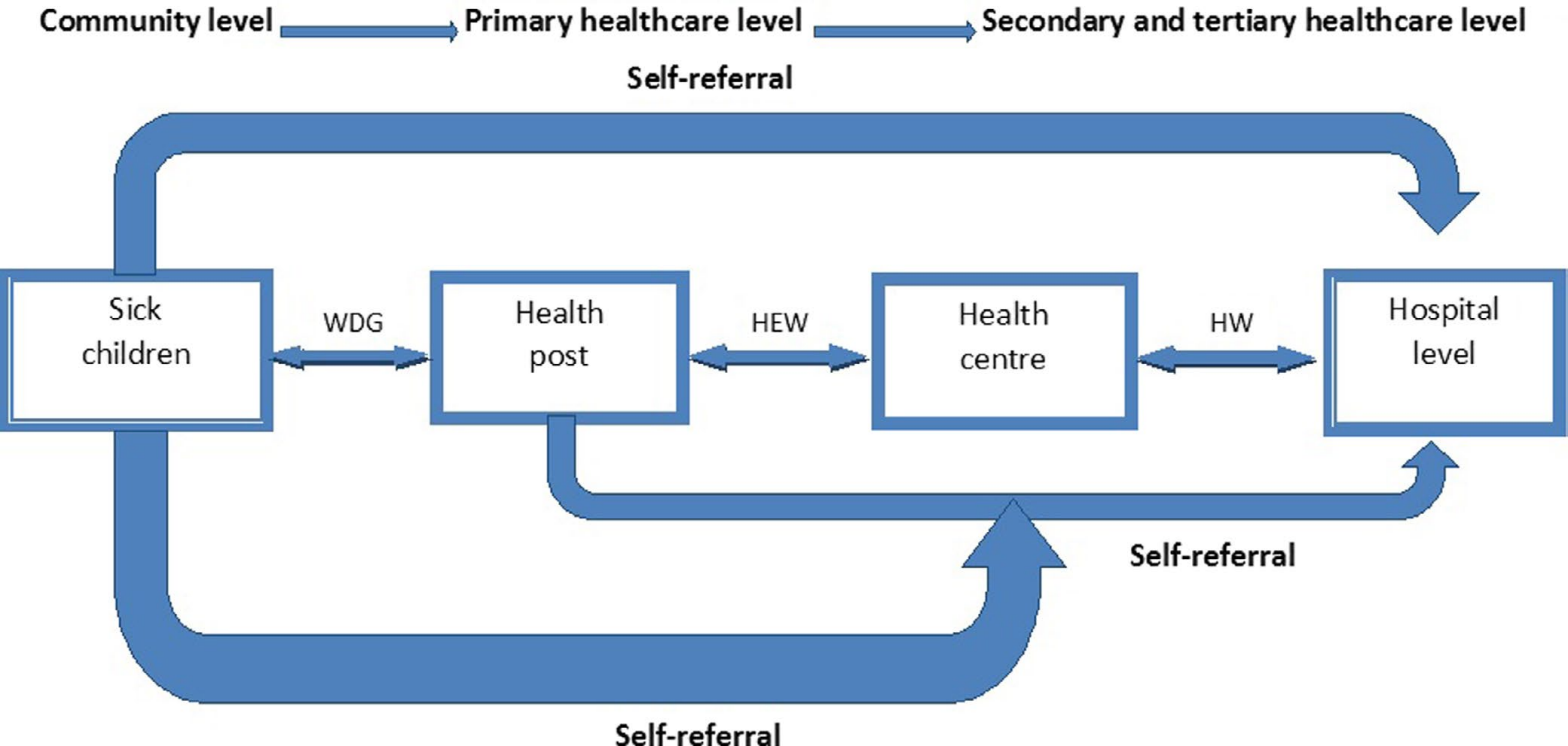
The Ethiopian referral pathways from primary to tertiary levels. HEW, health extension workers; HW, health workers; WDG, women's development group leaders

The survey was performed in 46 districts of the four largest regions of Ethiopia, namely Amhara, Southern Nations, Nationalities and Peoples, Oromia and Tigray regions. The referral care of sick children from the community, health post and health centre to higher health system levels in these study areas was assessed and related to referral guidelines of the different care programmes as mentioned above (Figure 1 and Table 1).

**Table 1:** Childhood diseases requiring urgent referral according to newborn and child programme guidelines in Ethiopia^16^

Disease	Sick infant 0‐59 d of age	Sick child 2‐59 mo of age
Very low birthweight or very preterm	Refer	
Possible serious bacterial infection or very severe disease	Refer	Refer
Severe jaundice	Refer	
Severe dehydration	Refer	Refer
Severe pneumonia	Refer	Refer
Severe persistent diarrhoea	Refer	Refer
Very severe febrile disease		Refer
Severe complicated measles		Refer
Mastoiditis		Refer
Severe anaemia		Refer
Complicated severe acute malnutrition		Refer

### 2.2 Study population and sampling

A two‐stage stratified cluster sampling was applied in the selected study areas. The first stage used lists of enumeration areas in the chosen districts from the 2007 Ethiopian Housing and Population Census as the sampling frame. The cumulative population across the study districts was calculated, and 200 enumeration areas were selected proportional to population size. Each enumeration area formed one cluster, and these clusters constituted the primary sampling units.

In the second stage, a systematic random sampling technique was used to select households within the selected enumeration area. For every cluster of 30 households, we interviewed one women's development group leader serving the cluster, one health extension worker at the health post serving the selected enumeration area, and staff at the corresponding health centre. Further, we reviewed the treatment registers at health posts and health centres. Interviews were also performed at the district health offices that provided support to the selected health providers and facilities.

The sample size was based on the requirement that the baseline and end‐line surveys should be able to assess changes in selected indicators of care‐seeking and treatment for a sick child based on household interviews (information not used in this report).

### 2.3 Data collection

Six of the enumeration areas were excluded from the study due to security concerns. A total of 60 trained health professionals and 14 supervisors collected the data in the 194 enumeration areas. The questionnaires had been adapted from existing large‐scale survey tools, such as the Demographic and Health Surveys and the Service Provision Assessment survey.^15^ The questionnaires were translated into three local languages (Amharic, Afan Oromo and Tigrigna), and pre‐tested. All data were collected using tablet computers.

We collected data on the background characteristics of the interviewees, such as knowledge of danger signs in newborns and under‐five children and their referral needs, as well as referral practices in the last 3 months.

The health facility data on the infrastructure available to facilitate the referral of sick children were collected. Data were also copied from health post and health centre treatment registers, including illness classifications, numbers referred, as well as reasons for their referrals in the 3 months preceding the survey.

Interviewers also collected information on the general profile of the district and other factors that might affect childhood referral care in their catchment area.

### 2.4 Data analysis

Descriptive statistics, that is frequencies, percentages and means, were computed to analyse selected demographic characteristics of the involved staff, their knowledge, and practices. Chi‐square tests were used to assess the association between referred under‐five children and their age, sex and illness classification. Census and Survey Processing System version 6.3.2 software (United States Census Bureau) was used for data entry and cleaning. Statistical analysis and tests were done using SPSS version 20 (IBM Corp).

### 2.5 Ethical approval

Ethical approval was secured from Hawassa University (IRB/199/10), Ethiopian Public Health Institute (SERO‐012‐8‐2016), and the London School of Hygiene & Tropical Medicine (protocol number 11235). Informed consent was obtained from all study participants before data collection.

## 3 RESULTS

Data on referrals were available from 187 women's development group leaders, 276 health extension workers based at 177 health posts, and 175 staff at 155 health centres. Information on the availability of ambulance services was retrieved from 46 district health offices. The referral of 225 sick young infants and 1259 sick children aged 2‐59 months was copied from registers at health posts. Similarly, the referral of 842 sick young infants and 1490 sick children 2‐59 months of age was transcribed from health centre registers.

### 3.1 Characteristics of study participants

Most women's development group leaders and health centre staff had served <4 years in their current position, whereas most health extension workers had worked for 9 years or more (Table 2). Nearly half (47%) of the women's development group leaders had no formal education, and a similar proportion of health extension workers had secondary level education. Three‐quarters of the health centre staff were college graduate nurses.

**Table 2:** Characteristics of study participants in the surveyed primary health care units

Characteristic	Levels	Women's development group leaders (n = 187)	Health extension workers (n = 276)	Health centre staff (n = 175)
Region	Amhara	69 (37%)	119 (43%)	75 (43%)
	Oromia	66 (35%)	97 (35%)	57 (33%)
	SNNP	23 (12%)	20 (7%)	17 (9.7%)
	Tigray	29 (16%)	40 (15%)	26 (15%)
Years of service	<4 y	132 (71%)	91 (33%)	136 (78%)
	4‐8 y	49 (26%)	69 (25%)	29 (17%)
	>9 y	6 (3.2%)	116 (42%)	10 (5.7%)

Note

Ethiopia, December 2016 to February 2017.

Abbreviation: SNNP, Southern Nations, Nationalities, and Peoples' region.

In the 12 months preceding the survey, half of the women's development group leaders had received training on danger signs of sick young infants (50%), also of children 2‐59 months (48%) and referral of sick young infants (46%).

Out of the surveyed health extension workers, 65% had received training for newborn care, and 83% on the management of childhood illnesses. Similarly, 47% and 74% of the health centre staff had been trained in these two fields, respectively.

The majority of health extension workers and health centre staff had adequate unprompted knowledge of urgent referral needs when sick young infants and children 2‐59 months of age presented with severe diseases (Table 3).

**Table 3:** Proportions of health extension workers and health centre staff who correctly identify referral needs for different child conditions

Conditions requiring urgent referral	Health extension workers (n = 276)	Health centre staff (n = 175)
Young infants <1.5 kg or gestational age <32 wk	230 (83%)	150 (86%)
Young infants with possible severe bacterial infections	239 (87%)	146 (83%)
Young infants with severe jaundice	207 (75%)	146 (83%)
Young infants with severe dehydration	212 (77%)	108 (62%)
Children 2‐59 mo of age with general danger signs	231 (84%)	146 (83%)

Note

Ethiopia, December 2016 to February 2017.

### 3.2 Referral

In the 6 months before the survey, 22% of the women's development group leaders visited at least one sick young infant, and 45% visited one or more sick children aged 2‐59 months. A majority (83%) of the leaders referred at least one sick young infant, and two‐thirds (67%) referred at least one older sick child. These figures correspond to a referral of 56% of the visited sick young infants, and 62% of the visited 2‐59‐month‐old sick children to the health extension workers.

The review of registers at health posts showed that more young infant boys than girls were seen. There were more visits of sick young infants during the first week of life at the health post, while at the health centre, the majority of young infants examined were 2‐4 weeks old (Table 4).

**Table 4:** Characteristics of all sick young infants seen in the 3 mo before survey and the last ten sick children aged 2‐59 mo

Characteristics	Levels	Sick infants 0‐59 d		Sick children 2‐59 mo	
		Health post (n = 225)	Health centre (n = 842)	Health post (n = 1259)	Health centre (n = 1490)
Sex	Boy	128 (57%)	480 (57%)	682 (54%)	819 (55%)
	Girl	97 (43%)	362 (43%)	577 (46%)	671 (45%)
Age	1st wk	98 (44%)	99 (12%)		
	2‐4 wk	77 (34%)	454 (54%)		
	5‐8 wk	50 (22%)	289 (34%)		
	2‐11 mo			432 (34%)	509 (34%)
	12‐23 mo			365 (29%)	407 (27%)
	24‐59 mo			462 (37%)	574 (39%)

Note

Data from health post and health centre registers. Ethiopia, December 2016 to February 2017.

At the health post, 16 per cent of the sick young infants examined, and 6% of sick children 2‐59 months were referred to higher levels. A higher proportion of boys was referred (Table 5). Possible severe bacterial infections in young infants were the leading cause of referral in this age group (61%, *P*‐value < .001), but only half of the children with that illness classification (22/44, 50%) were referred to the health centres. Children with local bacterial infection who are supposed to be treated at the health post were rarely referred (3/32, 9%).

**Table 5:** Referral of sick young infants 0‐59 d of age and sick children 2‐59 mo of age from health posts and health centres to higher levels

Characteristics	Levels	Health post referral				Health centre referral			
		Sick infants 0‐59 d		Sick children 2‐59 mo		Sick infants 0‐59 d		Sick children 2‐59 mo	
		Seen	Referred	Seen	Referred	Seen	Referred	Seen	Referred
Total		225	36 (16%)	1259	78 (6.2%)	842	51 (6%)	1490	22 (1.5%)
Sex	Boys	128	25 (20%)	682	42 (6.1%)	480	39 (8.1%)*	803	13 (1.6%)
	Girls	97	11 (11%)	557	36 (6.5%)	362	12 (3.3%)*	647	9 (1.4%)
Age	1st wk	98	13 (13%)			99	9 (9.1%)		
	2‐4 wk	77	14 (18%)			454	23 (5.1%)		
	5‐8 wk	50	9 (18%)			289	19 (6.6%)		
	2‐11 mo			432	22 (5.1%)			494	4 (0.8%)
	12‐23 mo			365	30 (8.2%)			402	6 (1.5%)
	24‐59 mo			439	26 (5.9%)			594	12 (2.0%)

Note

Ethiopia, December 2016 to February 2017.

* Chi‐square test for characteristic, *P*‐value < .01.

Among children aged 2‐59 months, suspected pneumonia (17/304, 6%) and fever when malaria had been excluded (17/166, 10%) dominated referral. All cases of severe pneumonia were referred to health centres, as per the guidelines.

In the follow‐up of the referred 36 young infants, the health extension workers noted that 19 had improved, one had not improved, and no feedback had been received for 16 young infants. Among the referred 78 sick 2‐59‐month‐old children 61 had improved, one had not improved, and feedback had not been received for the remaining 16 children.

A total of 6% of sick young infants and 2% of sick children 2‐59‐month‐old were referred from the health centres to higher levels. A higher proportion of sick young infant boys as compared to girls was referred (5% and 1%, respectively, *P*‐value < .01; Table 5).

Possible severe bacterial infection in young infants was the leading cause of referral to hospitals (69%, *P*‐value < .001), but only a third (35/111, 32%) of the sick young infants with that classification were referred. Children with local bacterial infection were rarely referred (2/180, 1%).

In the follow‐up of the referred 51 young infants from the health centre to the hospital, the staff noted that 15 had improved, and no feedback had been received for the remaining 36 young infants. Among the 22 referred sick 2‐59‐month‐old children ten had improved, and no feedback had been received for the remaining 12 children.

### 3.3 Referral logistics

We found that 23% of the women's development group leaders used paper forms for referral of sick children from the community to health posts. Further, only 31% of the health extension workers used forms when referring a child to a health centre for further care. Mobile phone signals were present in 77% of the surveyed health posts and 83% of the health centres. Mobile phones were reported to be used at more than half of the health posts during the last sick young infant referral. A functional ambulance or other transport for incoming referrals were available at a bit more than a third of the health centres. Most (94%) of the districts had at least one ambulance for the transportation of pregnant women. However, only three‐quarters made the ambulance available to sick children.

## 4 DISCUSSION

The women's development group leaders, who are not service providers, visited many sick children but referred only half of them to the health post. At the health post, the health extension workers referred only a fraction of the sick young infants and under‐five children to health centres. Even cases of possible severe bacterial infection, that is suspected sepsis, were frequently not referred. Referrals from health centre to hospital were rare. Despite this low level of referrals, the theoretical knowledge of referral needs was adequate among the health extension workers and health centre staff. The referral logistics were weak across the levels of the primary health care unit; referral forms were missing, mobile phone signals were not optimal, and ambulances or other transportation were often unavailable. Feedback on the referred cases from higher levels was often lacking.

The women's development group leaders are volunteers with a relatively low educational level. They visited families in their network with sick children and were supposed to refer sick children to the health extension worker at the health post. This group of health workers should be trained by the health extension workers but many leaders had not received this support. They also had insufficient training on referral of sick children. Previous studies have shown that the use of health services was on a higher level in areas where there was a well‐functioning women's development group network.^11^

A majority of the health extension workers in our study knew which sick young infants and under‐five children needed to be referred to a higher level, but this was not reflected in practice. A substantial number of severely sick infants and children were not referred as prescribed in the guidelines of the community‐based newborn care and the integrated community case management of childhood illnesses. A majority of the sick young infants (0‐59 days), who were examined at health posts, were in their first week of life. Those classified as having a very severe disease should be treated as potential sepsis cases and, according to the guidelines, get a first dose of gentamycin injection and oral amoxicillin and thereafter be urgently referred. Only half of these infants were managed in this way. Similar difficulties in achieving a well‐functioning referral system have been reported from other African countries. In a scale‐up of the integrated community case management in Malawi, only 55% were referred of those who should be selected according to guidelines.^17^

The leading causes of referral from health post of under‐five children were suspected pneumonia and fever when malaria had been excluded. Both conditions were expected to be managed at the health post. More boys than girls were seen and referred from the health posts. This can partly be due to the fact that male newborns tend to be more severely ill than girls,^18^, ^19^ but may also be related to the Ethiopian culture of male preference.^20^

As was the case with the health extension workers, the health centre staff had good knowledge of illnesses that require urgent referral. Very few children were, however, referred from health centres to higher levels. The leading illness classification among referred young infants was possible severe bacterial infection, but only a small fraction of those cases was referred. According to the guidelines of the integrated management of newborn and childhood illnesses, referral to hospital is recommended for this group of patients. Given that sepsis accounts for a third of neonatal deaths, the management of these cases and availability of referral are crucial.^21^

There could be several reasons for the lack of adherence to the referral guidelines. At the health worker level, despite knowing the need for referral they may act differently. It was beyond the scope of our study to explore the reasons behind this know‐do gap. In a study in Tanzania, only 25% of children classified to be severely ill at the primary care level were referred. In that setting, the health providers disagreed with the guidelines and considered management and treatment possible at the primary care unit.^22^ Parents might also refuse referral in spite of recommendations from the health worker.^23^ Mother's illness,^24^ distance to the higher‐level facility, travel cost, weather and perceived poor quality of care at the referral facility have all been shown to limit referral acceptance by parents.^13^, ^25^

Contrary to guidelines, many cases of non‐malaria fever were referred to health centres. Current guidelines state that these children with unclassified fever should be re‐evaluated by the health worker after 3 days. There is, however, evidence that the health extension workers safely can advise parents to return in case of no improvement.^26^ A modification of the treatment guidelines could reduce the workload on the health extension workers and maybe lower the number of unnecessary referrals. The referral of children with uncomplicated pneumonia from health posts to health centres could be due to lack of antibiotics at the lower level.^27^

A well‐functioning referral system is an essential part of the quality of care provided at health facilities.^28^ This study revealed insufficiencies in the logistics of referral. Referral forms were not used, although the lack of written referrals from women's development group leaders may be explained by their low educational level. Feedback loops of information and follow‐up were often absent. Although the mobile phone network in Ethiopia is rapidly expanding, connectivity was missing at many health posts. This limits the possible discussion and information exchange between levels in the health system. Ambulances for sick children were often not available. An Ethiopian health economics study indicated that an ambulance‐based referral system for emergency obstetric and newborn care should be highly cost‐effective.^29^

### 4.1 Strengths and limitations

This study was based on a survey in four of the largest regions in Ethiopia accounting for 80% of the population. The sample was selected to represent the chosen districts in these regions, where the Optimizing the Health Extension Program intervention was to be implemented. We have no reasons to believe that the selected districts differ in health systems characteristics from those of the four regions. Frequently, studies of sick child referral only cover one level in the health system.^24^, ^27^ We described referral from the community to health post, from health post to health centre, and from health centre to hospital level (Figure 1). We also describe the referral practices based on interviews with community volunteers and health workers at these levels and information from registers at health facilities supplemented the picture. The voice of the parents is, however, missing.^12^ Although we have referral information from community to hospital, we have not followed individual children to evaluate compliance and outcome. Recall bias is another limitation in our study for common reasons of adherence and lack of adherence to the treatment guidelines.

## 5 CONCLUSION

We have shown that the primary care referral system for sick newborns and children below the age of 5 years in four Ethiopian regions was not functioning well. The women's development group leaders are a resource that could provide more support to the health extension programme. In spite of good theoretical knowledge, the health workers at health centre and health posts did not adhere to referral guidelines. Lack of referral logistics, communication around patients and ambulances could partly explain these findings. Strengthening of the referral system is needed for an efficient primary health care system for children and further reduction of neonatal and deaths under the age of five in Ethiopia.

## CONFLICT OF INTEREST

The authors declare no conflict of interest.

## References

[1] GBD 2017 Mortality Collaborators . Global, regional, and national age‐sex‐specific mortality and life expectancy, 1950–2017: a systematic analysis for the Global Burden of Disease Study 2017. Lancet. 2018;392(10159):1684‐1735. 30496102 10.1016/S0140-6736(18)31891-9 PMC6227504

[2] Ruducha J , Mann C , Singh NS , et al. Articles How Ethiopia achieved Millennium Development Goal 4 through multisectoral interventions: a Countdown to 2015 case study. Lancet Global Health. 2015;5(11):e1142‐e1151. 10.1016/S2214-109X(17)30331-5 PMC5640803 29025635

[3] Ethiopian Public Health Institute (EPHI) [Ethiopia] and ICF . Ethiopia mini demographic and health survey 2019: key indicators. Rockville: EPHI and ICF 2019 https://www.dhsprogram.com/pubs/pdf/PR120/PR120.pdf. Accessed August 24, 2019.

[4] Marsh DR , Hamer DH , Pagnoni F , Peterson S . Introduction to a special supplement: evidence for the implementation, effects, and impact of the integrated community case management strategy to treat childhood infection. Am J Trop Med Hyg. 2012;87(Suppl 5):2‐5. 23136271 10.4269/ajtmh.2012.12-0504 PMC3748517

[5] Lulseged S . Integrated management of childhood illness: a review of the Ethiopian experience and prospects for child health. Ethiop Med J. 2002;40(2):187‐201. 12240581

[6] Marsh D , Nefdt R , Hazel E . Medical special issue: integrated community case management (iCCM) at scale in Ethiopia: evidence and experience. Ethiop Med J. 2014;52(Sup. 3): 47‐54. 25845086

[7] Miller NP , Amouzou A , Tafesse M , et al. Integrated community case management of childhood illness in Ethiopia: implementation strength and quality of care. Am J Trop Med Hyg. 2014;91(2):424‐434. 24799369 10.4269/ajtmh.13-0751 PMC4125273

[8] Federal Democratic Republic of Ethiopia Ministry of Health (FMOH): Health Sector Transformation Plan [Internet]. 2015 https://www.globalfinancingfacility.-org/-sites/gff_new/files/-Ethiopia-health-system-transformation-plan.pdf. Accessed August 15, 2019.

[9] Amouzou A , Hazel E , Shaw B , et al. Effects of the integrated community case management of childhood illness strategy on child mortality in Ethiopia: a cluster randomized trial. Am J Trop Med Hyg. 2016;94(3):596‐604. 26787148 10.4269/ajtmh.15-0586 PMC4775896

[10] Berhanu D . Community based newborn care in Ethiopia: Quality of CBNC programme assessment Midline Evaluation Report March 2017 [Internet]. 2017 https://ideas.lshtm.ac.uk/report/-cbnc-midline-eval-mar2017/. Accessed August 12, 2019.

[11] Yitbarek K , Abraham G , Morankar S . Contribution of women's development army to maternal and child health in Ethiopia: a systematic review of evidence. BMJ Open. 2019;9:e025937. 10.1136/bmjopen-2018-025937 PMC6538000 31122974

[12] Abrahim O , Linnander E , Mohammed H , Fetene N , Bradley E . A patient‐centered understanding of the referral system in Ethiopian primary health care units. PLoS ONE. 2015;10(10):e0139024. 26436759 10.1371/journal.pone.0139024 PMC4593586

[13] Newbrander W , Ickx P , Werner R , Mujadidi F . Compliance with referral of sick children: a survey in five districts of Afghanistan. BMC Pediatr. 2012;12(46):1471‐2431. 10.1186/1471-2431-12-46 PMC3422210 22540424

[14] Akbari A , Mayhew A , Al‐alawi MA , et al. Interventions to improve outpatient referrals from primary care to secondary care. Cochrane Database Syst Rev. 2014;(1): 1-50. 10.1002/14651858.CD005471.pub2 PMC4164370 18843691

[15] Central Statistical Agency (CSA) [Ethiopia] and ICF (2016) . Ethiopia: demographic and health survey 2016 [Internet]. 2016 https://www.dhsprogram.com/-publications/publication-FR328-DHS-Final-Reports.cfm. Accessed August 10, 2018.

[16] FMOH. Federal Ministry of Health, Ethiopia . Integrated management of newborn and childhood illness, part 1. Blended learning module for the health extension programme. 2011 https://www.open.edu/openlearncreate/pluginfile.php/71990/mod_resource/content/2/-IMNCI_Part_1_Final_Print-ready_March_2011_.pdf. Accessed August 15, 2019.

[17] Nsona H , Mtimuni A , Daelmans B , et al. Scaling up integrated community case management of childhood illness: update from Malawi. Am Soc Trop Med Hyg. 2012;87(Suppl 5):54‐60. 10.4269/ajtmh.2012.11-0759 PMC3748522 23136278

[18] Muenchhoff M , Goulder PJR . Sex differences in pediatric infectious diseases. J Infect Dis. 2014;209(Suppl 3):S120‐S126. 24966192 10.1093/infdis/jiu232 PMC4072001

[19] Gelaw YA , Biks GA , Alene KA . Effect of residence on mothers' health care seeking behavior for common childhood illness in Northwest Ethiopia: a community based comparative cross – sectional study. BMC Res Notes. 2014;7(705):1‐8. 25297952 10.1186/1756-0500-7-705 PMC4210615

[20] MDG Achievement Fund . UN Women: leave no women behind in advancing gender equality: promising practices ii: case studies from the millennium development goals Achievement Fund. 2013 http://www.unwomen.org/mdgf/B/Ethiopia_B.html. Accessed June 21, 2019.

[21] Mathewos B , Owen H , Sitrin D , et al. Community‐Based Interventions for Newborns in Ethiopia (COMBINE): cost‐effectiveness analysis. Heal Policy Plan. 2017;32:i21‐i32. 10.1093/heapol/czx054 28981760

[22] Walter ND , Lyimo T , Skarbinski J , et al. Why first‐level health workers fail to follow guidelines for managing severe disease in children in the Coast Region, the United Republic of Tanzania. Bull World Health Organ. 2009;87:99‐107. 19274361 10.2471/BLT.08.050740 PMC2636200

[23] Hailegebriel TD , Mulligan B , Cousens S , et al. Effect on neonatal mortality of newborn infection management at health posts when referral is not possible. Glob Heal Sci Pract. 2017;5(2):202‐216. 10.9745/GHSP-D-16-00312 PMC5487084 28611102

[24] Nalwadda CK , Waiswa P , Kiguli J , et al. High compliance with newborn community‐to‐facility referral in eastern Uganda: an opportunity to improve newborn survival. PLoS ONE. 2013;8(11):e81610. 24312326 10.1371/journal.pone.0081610 PMC3843697

[25] Nanyonjo A , Bagorogoza B , Kasteng F , Ayebale G , Makumbi F , Tomson G . Estimating the cost of referral and willingness to pay for referral to higher‐level health facilities: a case series study from an integrated community case management programme in Uganda. BMC Health Serv Res. 2015;15(347):1‐10. 26315661 10.1186/s12913-015-1019-5 PMC4551371

[26] Källander K , Alfvén T , Funk T , et al. Universal versus conditional day 3 follow‐up for children with non‐severe unclassified fever at the community level in Ethiopia: a cluster‐ randomised non‐inferiority trial. PLoS Med. 2018;946:1‐17. 10.1371/journal.pmed.1002553 PMC5903591 29664899

[27] Hailemariam S , Gebeyehu Y , Loha E , Johansson KA , Lindtjørn B . Inadequate management of pneumonia among children in South Ethiopia: findings from descriptive study. BMC Health Serv Res. 2019;7(19):1‐14. 10.1186/s12913-019-4242-7 PMC6595689 31242946

[28] World Health Organization . Standards for improving the quality of care for children and young adolescents in health facilities. Geneva: World Health Organization; 2018. Licence: CC BY‐NC‐SA 3.0 IGO.

[29] Accorsi S , Somigliana E , Solomon H , et al. Cost‐effectiveness of an ambulance‐based referral system for emergency obstetrical and neonatal care in rural Ethiopia. BMC Pregnancy Childbirth. 2017;17(220):1‐7. 28701153 10.1186/s12884-017-1403-8 PMC5506594

